# Sabirnetug (ACU193) Lowers CSF Levels of Synaptic Biomarkers in INTERCEPT‐AD Phase 1 Study in Early AD

**DOI:** 10.1002/alz.094967

**Published:** 2025-01-09

**Authors:** Erika N Cline, Elizabeth Johnson, Karen Sundell, Daniel Antwi‐Berko, Marleen JA Koel‐Simmerlink, Charlotte Teunissen, Eric Siemers, Hao Zhang, Maddelyn Hyland, Gopalan Sethuraman, Hugo Vanderstichele, June Kaplow, Robert A Dean, Jasna Jerecic

**Affiliations:** ^1^ Acumen Pharmaceuticals, Charlottesville, VA USA; ^2^ Amsterdam UMC, Amsterdam Netherlands; ^3^ Biomarkable, Gent Belgium; ^4^ Indiana University School of Medicine, Indianapolis, IN USA

## Abstract

**Background:**

Sabirnetug (ACU193) is a humanized IgG2 antibody targeting soluble, synaptotoxic amyloid β oligomers (AβOs). AβOs accumulate in Alzheimer’s disease (AD) and induce pre‐ and post‐synaptic changes, resulting in dendritic spine loss, neuronal degeneration, and release of synaptic proteins into the CSF. Recently, we reported that three administrations of sabirnetug in an early AD population (INTERCEPT‐AD Phase 1 study, NCT04931459) significantly lowered CSF levels of the post‐synaptic protein neurogranin as well as pTau181. Here, we present data on additional CSF synaptic biomarkers and AD plasma biomarkers measured in INTERCEPT‐AD.

**Method:**

INTERCEPT‐AD was a randomized, placebo‐controlled study with two parts: single ascending dose (SAD) randomized in a 6:2 ratio to sabirnetug (2, 10, 25, 60 mg/kg) or placebo and multiple ascending dose (MAD) randomized 8:2 to sabirnetug (three administrations at 10 or 60 mg/kg every 4 weeks [Q4W] or 25 mg/kg Q2W) or placebo. Biomarkers were measured blinded in CSF and EDTA‐plasma, before and after drug administration, by Amsterdam UMC.

**Result:**

CSF levels of vesicle‐associated membrane protein 2 (VAMP2), a protein involved in pre‐ and post‐synaptic vesicle trafficking, were significantly normalized (lowered) in sabirnetug treated participants relative to placebo in all three MAD cohorts. Levels of the pre‐synaptic protein neuronal pentraxin 2 (NPTX2), which acts on post‐synaptic excitatory synapses, regulates complement activity, and lowers with cognitive decline, trended lower with sabirnetug than placebo. Plasma levels of GFAP, pTau181, and pTau217 trended towards normalization (lowering) of AD‐dependent changes in the 60 mg/kg Q4W cohort.

**Conclusion:**

In INTERCEPT‐AD, three administrations of sabirnetug lowered CSF levels of both pre‐ and post‐synaptic proteins, consistent with sabirnetug’s proposed mechanism of action to inhibit AβO synaptic binding. VAMP2 appeared most sensitive to sabirnetug in this study, lowering significantly in all three MAD cohorts; other markers previously reported to have a statistically significant response to sabirnetug ‐ neurogranin and pTau181 ‐ did so at the highest dose. No statistically significant changes in plasma biomarkers were observed in this short study. Long‐term changes in biomarker levels and their relationship to clinical efficacy will be evaluated in the 18‐month ALTITUDE‐AD phase 2 study of sabirnetug beginning in the first half of 2024.

